# Programming temporal stiffness cues within extracellular matrix hydrogels for modelling cancer niches

**DOI:** 10.1016/j.mtbio.2024.101004

**Published:** 2024-02-16

**Authors:** Gretel Major, Minjun Ahn, Won-Woo Cho, Miguel Santos, Jessika Wise, Elisabeth Phillips, Steven G. Wise, Jinah Jang, Jelena Rnjak-Kovacina, Tim Woodfield, Khoon S. Lim

**Affiliations:** a Department of Orthopaedic Surgery and Musculoskeletal Medicine, Centre for Bioengineering & Nanomedicine, University of Otago, Christchurch, New Zealand; b Pohang University of Science and Technology (POSTECH), Pohang, South Korea; c Applied Materials Group, School of Medical Sciences, University of Sydney, Sydney, Australia; d Mackenzie Cancer Research Group, Department of Pathology and Biomedical Science, University of Otago, Christchurch, New Zealand; e Graduate School of Biomedical Engineering, University of New South Wales, Sydney, Australia; f Tyree Institute of Health Engineering, University of New South Wales, Sydney, NSW, 2052, Australia; g Light-Activated Materials Group, School of Medical Sciences, University of Sydney, Australia

## Abstract

Extracellular matrix (ECM) stiffening is a common occurrence during the progression of many diseases, such as breast cancer. To accurately mimic the pathophysiological context of disease within 3D *in vitro* models, there is high demand for smart biomaterials which replicate the dynamic and temporal mechanical cues of diseased states. This study describes a preclinical disease model, using breast cancer as an example, which replicates the dynamic plasticity of the tumour microenvironment by incorporating temporal (3-week progression) biomechanical cues within a tissue-specific hydrogel microenvironment. The composite hydrogel formulation, integrating adipose-derived decellularised ECM (AdECM) and silk fibroin, was initially crosslinked using a visible light-mediated system, and then progressively stiffened through spontaneous secondary structure interactions inherent between the polymer chains (∼10–15 kPa increase, with a final stiffness of 25 kPa). When encapsulated and cultured *in vitro*, MCF-7 breast cancer cells initially formed numerous, large spheroids (>1000 μm^2^ in area), however, with progressive temporal stiffening, cells demonstrated growth arrest and underwent phenotypic changes resulting in intratumoral heterogeneity. Unlike widely-investigated static mechanical models, this stiffening hydrogel allowed for progressive phenotypic changes to be observed, and fostered the development of mature organoid-like spheroids, which mimicked both the organisation and acinar-structures of mature breast epithelium. The spheroids contained a central population of cells which expressed aggressive cellular programs, evidenced by increased fibronectin expression and reduction of E-cadherin. The phenotypic heterogeneity observed using this model is more reflective of physiological tumours, demonstrating the importance of establishing temporal cues within preclinical models in future work. Overall, the developed model demonstrated a novel strategy to uncouple ECM biomechanical properties from the cellular complexities of the disease microenvironment and offers the potential for wide applicability in other 3D *in vitro* disease models through addition of tissue-specific dECM materials.

## Graphical abstract

**Figure:**
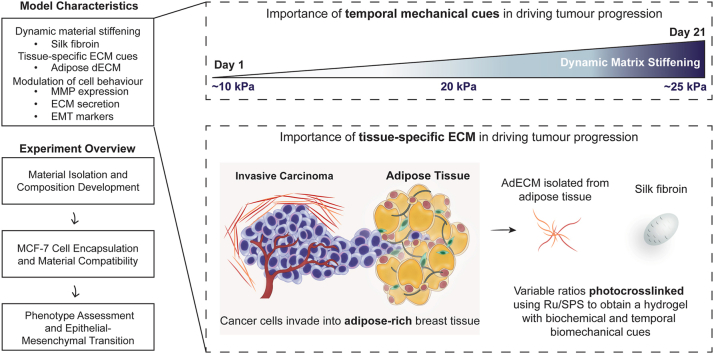

## 1 Introduction

**Fig. 1:**
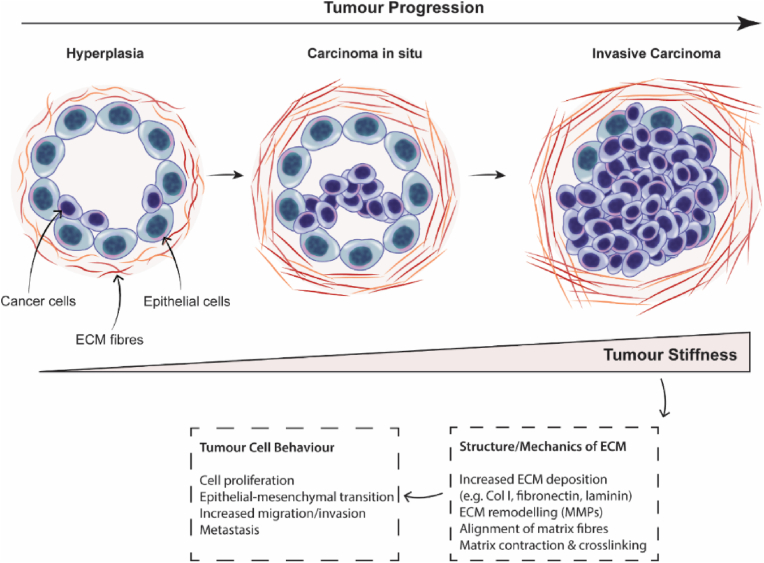
Schematic of tumour progression, highlighting dynamic changes in the structure and mechanics of the extracellular matrix (ECM) which modulate tumour cell behaviour. Throughout tumour progression, changes in deposition, alignment, composition and crosslinking of the ECM result in increasing tumour stiffness.

Temporal extracellular matrix (ECM) stiffening commonly occurs during disease progression in a range of medical indications, including pulmonary fibrosis, neurodegenerative conditions, and cancers (predominantly in pancreatic ductal adenocarcinoma and breast cancer) [1]. For example, in breast cancer the mechanical properties of native breast tissue are ∼3 kPa, while breast tumour ECM is considerably stiffer at ∼10–42 kPa depending on disease stage [2]. This indicates that as disease progresses, breast tumour tissue exhibits elastic moduli which are between 3- and 13-fold higher than native tissue [2], and demonstrates a shear stiffness that is ∼400% higher than surrounding breast tissue [3]. The ECM niche provides biochemical cues and structural support to cells, and regulates cell fate by dictating cell architectures, growth patterns, survival and motility [4]. Ongoing interactions between tumour cells and the tumour microenvironment throughout disease progression result in ECM fibre alignment, increased ECM deposition (collagens, laminins, fibronectin, proteoglycans etc.), increased crosslinking density and compositional changes within the ECM, which all contribute to increasing tissue stiffness as disease progresses (Fig. 1) [[4], [5], [6], [7], [8], [9], [10], [11], [12]]. Considering that ECM stiffening plays a key role in the evolution of disease (e.g., tumours) by orchestrating cellular programs through mechano-transduction pathways, it is crucial that preclinical models mimic the mechanical environment of evolving tissues to ensure that a representative cell phenotype is studied [13,14].

For disease modelling *in vitro*, it is now commonly accepted that two-dimensional (2D) models are not suitable for replicating the ECM, cell-cell interactions or the spatiotemporal complexity of diseased microenvironments (e.g., ECM arrangement, oxygen and growth factor gradients) and therefore three-dimensional (3D) models are under rapid development [15,16]. To reproduce simplified disease microenvironments, contemporary models have focused on the encapsulation of cells within a hydrated polymeric matrix, mimicking the 3D environment and structure of native ECM, allowing for the establishment of typical cell-cell and cell-ECM interactions [[17], [18], [19], [20], [21], [22], [23]]. Hydrogel models are particularly appealing as they provide not only the structural template, but the physicochemical cues (e.g., nutrient, metabolite and oxygen gradients) and mechanical cues which drive physiological phenotypes [16]. For example, compared with traditional 2D models, breast cancer cells grown in hypoxic 3D hydrogels demonstrate a similar upregulation of estrogen receptor alpha as seen in native tumours [24]. However, despite the diverse assortment of biomaterials utilised for preclinical modelling of the ECM in disease, there is a clear dichotomy between tunability and physiological relevance to the disease microenvironment.

While existing synthetic and semi-synthetic materials (e.g., polyethylene glycol (PEG), and peptide- and gelatin-based hydrogels) allow control over the structural hierarchy and physico-chemical properties (e.g., mechanics, porosity, degradation profiles) of preclinical models [21,[25], [26], [27], [28], [29], [30]], natural materials (e.g., Matrigel™, collagen I, alginate and fibrin) better replicate the pathophysiological context of disease and better support the growth of *in vitro* cellular organoids/spheroids [17,20,[31], [32], [33], [34], [35], [36], [37], [38], [39]]. As the organisation and stoichiometric composition of the ECM varies widely between tissues [5,40], ultimately dictating cell behaviours (e.g., migration patterns), new efforts have focused on the development of more complex natural materials from specific tissues, i.e., decellularised ECM (dECM) materials. These materials provide increased capacity to replicate the complexity and specificity of disease niches and have allowed for more sophisticated *in vitro* studies to be achieved [[41], [42], [43], [44], [45], [46]]. In breast cancer, adipose tissue is the main component of the breast and consists of an ECM of organised collagen VI and elastin fibres, and a high proportion of proteoglycans, which allow for enhanced flexibility and elasticity [47,48]. Breast cancer cells grown in adipose-derived dECM undergo phenotypic changes which imitate the cellular organisation and mesenchymal markers (N-cadherin and vimentin) observed in xenograft models [43], and exhibit enhanced features of tumour progression (cell growth, migration and chemoresistance) [44]. However, while the use of dECM from breast or adipose tissue has provided more precise ECM composition and collagen fibril ultrastructure to disease models, the low mechanical stiffness (∼0.1–5 kPa) of natural materials, and the limited capacity to tune the structural, mechanical and degradation profiles of the resulting hydrogel, mean that they are restricted in their ability to provide appropriate mechanical cues which mimic the disease microenvironment [13,[49], [50], [51]]. Attempts to improve the properties of dECM hydrogels have explored chemical crosslinking [52] and photocrosslinking strategies [53] to establish crosslinked networks, or forming composite hydrogels with other natural or synthetic materials to add structural support [[54], [55], [56], [57]]. However, there is still a clear trade-off between replicating the composition of the ECM, and providing tunability and control over material properties, highlighting the need for enhanced tissue-specific materials for modelling disease microenvironments.

To study the effects of static ECM mechanics on disease progression in *in vitro* models, most studies focus on tuning the initial hydrogel properties to achieve distinct hydrogels of variable static stiffness (through modulating the density of polymer or number of crosslinks formed), and then transferring cells between matrices or comparing cellular phenotype in side-by-side hydrogels [13,31,[58], [59], [60], [61], [62], [63], [64]]. Early work utilised hydrogel systems to understand the effect of matrix stiffness on integrin adhesions, cellular organisation and growth patterns [65]. Breast cancer spheroids grown in soft (2 kPa) Gel-MA/collagen hydrogels initially demonstrate greater invasive properties, while spheroids grown in stiff (12 kPa) hydrogels activate intracellular metastatic programs which caused fibronectin deposition and remodelling of the tumour microenvironment to facilitate invasion [59]. A common strategy used to generate *in vitro* disease models with similar mechanical properties to native tissues involves using human dECM from diseased and fibrotic tissues, with differing native tissue mechanical properties [46,66,67]. These models result in static hydrogels which closely resemble native tissue mechanics from distinct diseased states. Fewer attempts have been made to incorporate temporal stiffening cues within these 3D models using on-demand or stimuli-responsive technologies. Addition of sugar threose to collagen gels for 24 h, results in a 1.8-fold increase in collagen I stiffness [68], while alginate can be temporally stiffened from 0.2 kPa to 2 kPa with addition of calcium-loaded liposomes and near-infrared light [69]. However, these dynamic materials require external stimuli (such as sugars and light irradiation) which may alter cell behaviour and do not demonstrate the scale of mechanical changes measured in diseased states (such as the ∼14-fold increase measured in native breast tumours) [2]. Furthermore, all of these approaches occur via a single-step switch rather than dynamically over time as seen biologically during tumour progression. Clearly, modelling the dynamic and temporal changes in the mechanical landscape of diseased microenvironments remains an unresolved challenge.

**Fig. 2:**
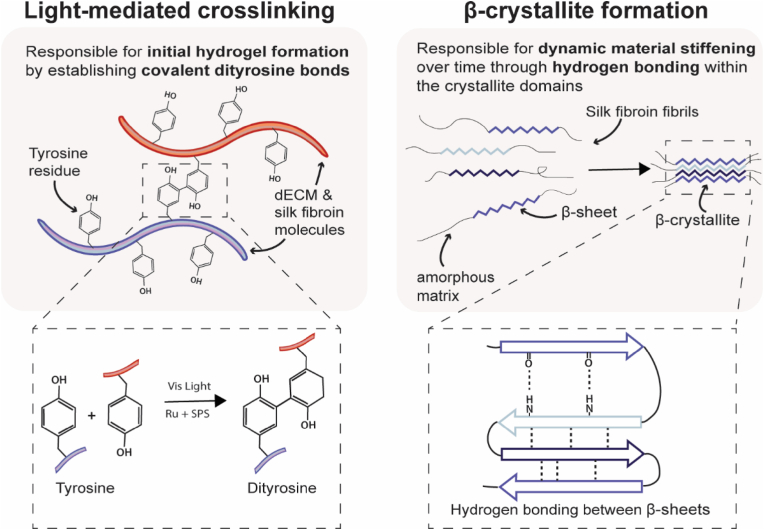
Crosslinking methods to form AdECM and silk hydrogels. Visible light-mediated crosslinking with ruthenium (Ru) and sodium persuflate (SPS) forms dityrosine bonds between materials, and spontaneous hydrogen bonding forms β-sheets between silk fibroin fibrils over time without external stimuli.

Silk fibroin, from *Bombyx mori* silkworms, is a cytocompatible and biodegradable biomaterial used in tissue engineering because of its widely tunable physicochemical properties [70,71]. Silk hydrogels can be fabricated through the formation of dityrosine bonds between polymer chains using a variety of crosslinking techniques, including enzymatic, Fenton reaction and photopolymerisaton [[72], [73], [74], [75], [76], [77], [78]]. Silk materials also undergo spontaneous protein structural transformations in the absence of chemical crosslinkers, due to random coil to β-sheet formation through hydrogen bonding between hydrophobic regions, resulting in hydrogels with dynamic stiffening and increased crystallinity over time (Fig. 2) [71,78,79]. Due to its favourable material properties, silk fibroin has also been incorporated with other biomaterials (e.g., synthetic compounds, dECM, resilin, gelatin, hyaluronic acid), to form mechanically- and structurally-enhanced materials [57,[78], [79], [80], [81], [82]]. Furthermore, as silk fibroin is relatively bioinert without inherent cell binding motifs, the abundant tyrosine residues present within the primary protein structure (5.3% of the amino acid composition) can be utilised to covalently and tunably biofunctionalise the material with ECM proteins for improved cell attachment and function [[82], [83], [84], [85]]. While silk fibroin does not mimic the ECM composition of the tumour microenvironment, its dynamic stiffening properties and bioinert state provide a unique opportunity to uncouple and mimic ECM mechanics during tumour progression without reliance on remodelling stromal cells.

The primary objective of this study was to develop a smart biomaterial formulation which can be programmed to accurately reflect the dynamic ECM stiffness of breast tissue throughout tumour progression, and to investigate how these temporal ECM changes modulate the behaviour of breast tumour cells in 3D *in vitro* culture. The specific aims were to 1) isolate and characterise adipose-derived dECM (AdECM), 2) develop a composite hydrogel formulation through photocrosslinking of AdECM and silk fibroin which undergoes temporal dynamic stiffening over culture time, 3) encapsulate luminal MCF-7 breast cancer cells within the hydrogels and verify survival, and 4) evaluate changes in cell phenotype and behaviour longitudinally across 3D *in vitro* culture in response to the programmed dynamic stiffening.

We demonstrate the development of a simple preclinical model of breast cancer using a composite material formulation that mimics the adipose-rich ECM of native breast tissue as well as the temporal mechanical changes which occur throughout tumour progression. MCF-7 cells grown in this composite hydrogel formulation demonstrate a reduction in epithelial markers, changes to the abundance of matrix remodelling enzymes and altered expression of ECM proteins without the need for complex cell cocultures.

## 2 Materials and methods

### 2.1 Isolation and characterisation of adipose-derived dECM

#### 2.1.1 Adipose tissue collection and decellularisation

Porcine adipose tissue samples used in this study were collected from an abattoir, with ethical approval from the Catholic University of Korea Institutional Review Board. The adipose tissue was decellularised as described previously [86]. Briefly, the tissue was cut into ∼3 mm^3^ pieces and then treated with 0.5% (w/v) sodium dodecyl sulphate (SDS) (to remove cellular contents), and isopropanol (to remove lipid contents), under continual agitation at 4 °C. The processed tissue was sterilised in 0.1% (v/v) peracetic acid in 4% (v/v) ethanol, and then lyophilised and milled into a fine powder, to obtain the adipose-derived dECM (AdECM).

#### 2.1.2 Preparation of AdECM

AdECM biomaterials were prepared at 3% (w/v) by digesting the AdECM powder in 0.5 M acetic acid with pepsin at 10% w/w for 72 h. After solubilisation of AdECM, the solution was centrifuged and stored at 4 °C until further experimentation. Directly before use, the AdECM biomaterial was neutralised to pH 7.4 with 10 M sodium hydroxide at 4 °C.

#### 2.1.3 Histological evaluation of AdECM

To visually evaluate effective decellularisation, AdECM was compared histologically with native adipose tissue. Haematoxylin and eosin (H&E) staining was used to evaluate tissue structure and presence of nuclei, while presence of collagens and glycosaminoglycans was evaluated using Masson's trichrome and Alcian blue staining, respectively. Native adipose tissue and AdECM were fixed in 4% paraformaldehyde and cryosectioned into 30 μm slices. The sectioned tissues were stained using commercial kits of H&E (Abcam, ab245880), Masson's trichrome (Abcam, ab150686), and Alcian blue (Abcam, ab150662) as per the manufacturers' instructions.

#### 2.1.4 Biochemical evaluation of AdECM

To quantify decellularisation, DNA, collagen and glycosaminoglycan contents were quantified using biochemical assays. To measure DNA content, equal weights of lyophilised native tissue and AdECM were solubilised in 125 μg/mL papain solution at 60 °C overnight. DNA content was quantified using the Hoechst 33258 fluorometric assay [87]. Conventional hydroxyproline [88] and dimethylmethylene blue [89] assays were used to quantify collagen and glycosaminoglycan content, respectively. The absorbance was measured at a wavelength of 550 nm for collagen and at 492 nm for glycosaminoglycans using a microplate reader.

#### 2.1.5 Proteomic analysis of AdECM

To characterise the composition of ECM proteins present within the AdECM, mass spectrometry (MS) analyses were conducted as described previously [90]. For sample preparation, AdECM was solubilised and digested into tryptic peptides for downstream analysis. The AdECM was reduced in 10 mM dithiothreitol (DTT) in the dark for 1h, followed by alkylation in 30 mM of 2-chloroacetamide for 1 h. To stop the reaction, 20 μL of 200 mM DTT was added to the sample, followed by incubation at 37 °C for 10 min. The reduced and alkylated sample was dissolved in 25 mM ammonium bicarbonate and subsequently treated with 2 μg of trypsin at 37 °C for 12 h. To ensure complete digestion, a further 1 μg of trypsin was added to the sample and incubated at 37 °C for 6 h. The sample was desalted using a C18 solid-phase extraction pipette tip and the resulting sample lyophilised. Finally, the dried sample was dissolved in 10 μL of 5% acetonitrile and 0.1% formic acid.

The mass spectrometry analysis was performed using a high-performance liquid chromatograph (Waters® nanoAcquity, USA) combined with an electrospray-ionisation FT/ion-trap mass spectrometer (LTQ Orbitrap Velos, Thermo Fisher Scientific, USA). The peptides were separated using a 50 × 365-μm fused silica capillary micro-column packed with 15 cm of 5 μm diameter C18 beads, followed by a 5 cm trap column (75 × 365 μm, 5-μm diameter C18 beads). The peptides were eluted at a constant flow rate of 0.2 μm/min, using a linear gradient from 5% to 50% of acetonitrile dissolved in 0.1% formic acid. A full-range mass spectral scan was obtained, and the MS/MS spectra raw data was analysed using SEQUEST version 1.2 against the porcine protein UniProt database. To assess the ECM proteins present within the AdECM, the dataset was further annotated using MatrisomeDB [91].

### 2.2 Isolation of silk fibroin

Silk fibroin solution was isolated from *Bombyx mori* silkworm cocoons as previously described [71,78]. To remove sericin from the silk fibroin fibres, silk cocoons (Tajima Shoji, Japan) were cut into small pieces and boiled in 0.02 M sodium carbonate solution (5 g cocoons per 2 L of Na_2_CO_3_). The degummed silk fibroin fibres were dissolved in 9.3 M lithium bromide at 25% w/v for 4 h at 60 °C. The silk fibroin solution was then dialysed against deionised water using a cellulose membrane (14 kDa cut-off) for 3 days. The concentration of silk was calculated by weighing the dialysed silk solution before and after lyophilisation.

### 2.3 Preparation of AdECM/Silk fibroin composite hydrogels

To obtain tissue-specific materials which undergo temporal dynamic stiffening throughout culture, a composite AdECM/silk fibroin hydrogel was formed by blending AdECM and silk fibroin (Table 1; Fig. S1). The composite gel precursors were photocrosslinked into hydrogels using a visible light-mediated crosslinking technique [53,78,92]. The gel precursors were mixed with photoinitiators; tris(2,2-bipyridyl) dichlororuthenium(II) hexahydrate (Ru) and sodium persulfate (SPS) at 0.5 mM and 5 mM respectively, and then photocrosslinked using visible light (OmniCure® S1500 with a Rosco IR/UV filter, 400–450 nm) at 30 mW/cm^2^ for 3 min in cylindrical (h = 1 mm, Ø = 5 mm) silicone moulds. To induce immediate beta-sheet formation (rather than spontaneous time-dependent transition), acellular samples in some experiments were incubated in 80% methanol overnight before further analysis [93,94].

**Table 1:** Composite AdECM/silk material formulations.

Composite Material	Final Concentration (% wt/wt)	
	AdECM	Silk Fibroin
2% AdECM	2	0
2% AdECM, 0.5% silk fibroin	2	0.5
0.5% AdECM, 2% silk fibroin	0.5	2
2% silk fibroin	0	2

### 2.4 Quantification of β-sheet crystallinity using FTIR-ATR

To induce β-sheet formation in the absence of cells, samples of each material composition were treated with 80% methanol overnight and lyophilised before determination of β-sheet crystallinity by Fourier transform spectroscopy in attenuated total reflectance mode (FTIR-ATR) [78]. Infrared spectra were collected by averaging a total of 64 scans at a spectral resolution of 4 cm^−1^ within the wavenumber range of 500–4500 cm^−1^. Amide I bands (1585–1710 cm^−1^) were deconvoluted using a Fourier self-deconvolution (FSD) algorithm by adopting Gaussian line profiles. The relative contribution of each secondary structure was determined by integration of the corresponding fitted peaks obtained from FSD calculations. The position of each peak was further confirmed by double differentiation of the amide I envelopes and peak assignment was as follows: side chains (1595–1605 cm^−1^); β-sheets (1610–1628 cm^−1^ and 1697–1704 cm^−1^); random coils (1635–1650 cm^−1^); alpha-helix (1652–1660 cm^−1^), and beta-turns (1663–1695 cm^−1^) (Fig. S2). A nonlinear least-square convergence criterion monitored the error between the original and deconvoluted spectra, imposing relative errors below 10^−6^. The contribution of β-sheets was determined by calculating the relative areas of the corresponding peaks in the area-normalised deconvoluted amide I profile (Fig. S1).

### 2.5 Compression testing of composite hydrogels

Unconfined compression testing was performed on a Physica MCR301 rheometer (Anton Paar, Germany), fitted with an optical Peltier plate setup and plate–plate geometry (25 mm diameter). Tripilicate samples from each material composition ( ± methanol treatment and ± cells) were tested at day 1, 7, 14 and 21. A constant compressive rate of 0.01 mm/s was applied to samples, with data output in the form of axial force (N) and gap (mm). A stress/strain curve for each replicate hydrogel was obtained and the compressive modulus (kPa) calculated from the linear region (5–15% strain) of the stress/strain curve.

### 2.6 Breast cancer cell encapsulation

#### 2.6.1 Cell culture and material encapsulation

MCF-7 breast cancer cells were purchased from the American Type Culture Collection and were routinely cultured in Dulbecco's modified eagle media (DMEM) with high-glucose (4,500 mg/L), 10% foetal bovine serum (FBS) and 1% penicillin/streptomycin. MCF-7 cells were encapsulated within composite AdECM/silk hydrogels (Table 1), at 2 million cells/mL in cylindrical moulds as described above. All encapsulated hydrogels were routinely cultured in 48 well-plates in DMEM supplemented with 10% FBS and 1% penicillin/streptomycin for 21 days, with media refreshed every three days. At day 1, 7, 14 and 21 samples were collected for compression testing, cellular analyses and histological evaluation. All experiments in this study were carried out using cell lines that were passaged <10 times.

### 2.7 Cell phenotype and behavioural assessment

#### 2.7.1 Cell viability

The cytocompatibility of the photocrosslinked composite AdECM/silk fibroin hydrogels was determined after one day using live/dead staining [95]. Samples were washed in PBS and stained with 1 μg/mL calcein-AM and 1 μg/mL propidium iodide for 15 min at RT. The samples were washed with PBS and imaged on a fluorescence microscope (Zeiss Axioimager Z1 microscope, Germany). Image analysis was undertaken using ImageJ, with percentage of viable cells calculated.

#### 2.7.2 Metabolic activity quantification

To track cell proliferation over time, the metabolic activity of samples was measured on day 1, 7, 14 and 21. Briefly, each sample was incubated with media containing 10% alamarBlue® solution for 3 h at 37 °C (Thermo Fisher Scientific, USA). The fluorescence was measured at an excitation wavelength of 530 nm and emission wavelength of 590 nm (according to the manufacturer's instructions).

#### 2.7.3 Histology and immunohistochemistry

At each time point (day 1, 7, 14 and 21), cultured samples were placed into 10% neutral buffered formalin overnight. Fixed samples were then incubated with OCT compound and sectioned at a thickness of 5 μm using a Leica CM1860 UV cryostat (Leica Biosystems, USA). Overall changes to matrix and spheroid structure was evaluated using H&E staining (Richard-Allen Scientific, USA). To assess tumour progression, immunohistochemistry staining was completed for epithelial-mesenchymal-transition markers and ECM components. For E-cadherin (*24E10,* Cell Signalling Technology), N-cadherin (*D4R1H,* Cell Signalling Technology)*,* vimentin (*D21H3,* Cell Signalling Technology), fibronectin (*E5H6X,* Cell Signalling Technology), laminin (*ab11575,* Abcam) and collagen I (*ab34710,* Abcam) staining, endogeneous peroxidase activity was blocked by incubating slides in 0.3% H_2_O_2_ for 10 min, followed by aspecific protein blocking with 5% bovine serum albumin in PBS for 30 min at RT and antigen retrieval by boiling samples at 95 °C in 10 mM citrate buffer (pH 6) for 20 min. All primary antibodies were incubated with samples overnight at 4 °C with rabbit IgG isotypes used as negative controls at concentrations matched with those of the primary antibodies (Table 2). For HRP detection, slides were then incubated with a Goat Anti-Rabbit IgG H&L (HRP) (*ab205718*, Abcam) secondary antibody at 1:5000 (400 ng/mL) for 1 h at RT. Slides were visualised by 3,3′-diaminobenzidine (DAB) oxidation and counterstained with haematoxylin to visualise cell nuclei. All stained slides were washed, dehydrated in graded ethanol (70–100% EtOH) and mounted in DPX. Slides were viewed and imaged at 20 × using an Aperio CS2 Slide scanner. Three sections per sample were stained and positive cells were analysed using Adobe Photoshop C6 and ImageJ software.

**Table 2:** Immunohistochemistry antibody concentrations and isotype dilutions.

Primary Antibody	Antibody Dilution	Final Concentration	Isotype Dilution
E-cadherin (*24E100)*	1:200	0.27 μg/mL	1:6204
N-cadherin (*D4R1H)*	1:100	1.42 ng/mL	1:1180
Vimentin (*D21H3)*	1:200	0.23 μg/mL	1:7283
Fibronectin (*E5H6X)*	1:100	1 μg/mL	1:1675
Laminin (*ab11575)*	1:50	0.01 μg/mL	1:167500
Collagen I (*ab34710)*	1:100	0.01 μg/mL	1:167500

#### 2.7.4 Matrix metalloproteinase antibody array

The secretion of matrix remodelling enzymes was measured using an antibody membrane array (*ab169816*, Abcam). To quantify the secreted proteins, media samples (refreshed 24 h prior) were collected on day 1 and 21 and were stored at −20 °C until the array was executed. For each time point, duplicate samples were pooled from three experiments and incubated with the membrane as per the manufacturer's instructions.

#### 2.7.5 Statistical analysis

All experiments were performed in triplicate for each crosslinking condition and repeated across three individual experiments. The results are expressed as mean ± standard error of the mean. Using GraphPad Prism 9 (GraphPad Prism 9, USA), groups of data were compared by one-way ANOVA or two-way ANOVA, with a P value of <0.05 considered statistically significant. Where relevant, post hoc Tukey's HSD tests were used for pairwise comparisons following ANOVA.

## 3 Results and discussion

**Fig. 3:**
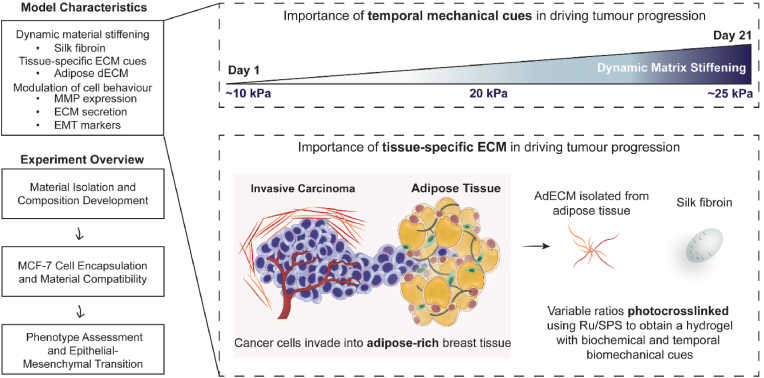
Overview of the breast cancer model characteristics and experimental workflow. The AdECM and silk fibroin composite hydrogel contains two natural materials which provide tissue-specific ECM cues and temporal biomechanical cues, which modulate tumour cell behaviour. The developed hydrogel assesses the importance of matrix stiffening and ECM composition in driving tumour progression without coculturing multiple cell types.

In this study we describe the development of a composite biomaterial formulation which incorporates temporal dynamic stiffening cues into a 3D *in vitro* disease model and uncouples these ECM mechanical cues from the complex cellular signalling which occurs in traditional coculture preclinical models. Using breast cancer as an example model system, a composite hydrogel formulation was developed which incorporates AdECM and silk fibroin to mimic the native breast ECM composition as well as the dynamic temporal stiffening of tumours, respectively (Fig. 3). The approach taken involved 1) isolating AdECM from porcine tissue and undertaking proteomic characterisation, 2) developing an AdECM/silk fibroin hydrogel through photocrosslinking with Ru/SPS and characterising changes in the mechanical profile longitudinally, 3) encapsulating luminal MCF-7 breast cancer cells in the hydrogels and assessing spheroid growth patterns, and 4) evaluating the phenotypic changes which occurred over three weeks of 3D *in vitro* culture in response to the programmed temporal stiffening.

### 3.1 Isolation and characterisation of AdECM

**Fig. 4:**
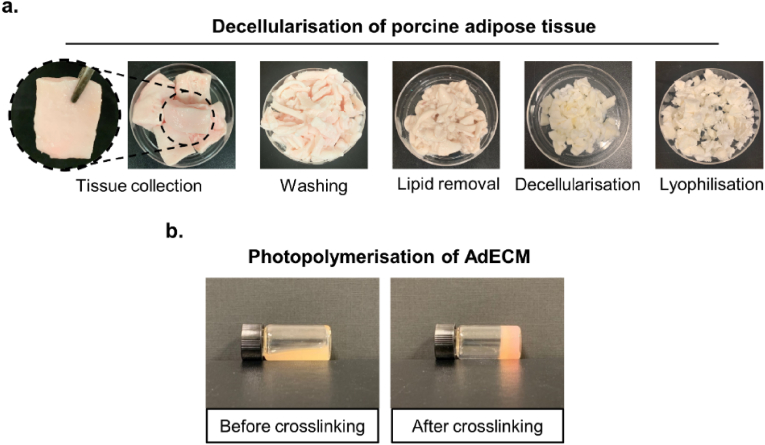
Development of AdECM from porcine adipose tissue. **a.** Visual overview of the decellularisation protocol involving step-wise removal of cellular material and lipids using SDS and isopropanol, respectively. The resulting decellularised material was lyophilised and ground to a powder before digestion with pepsin. **b.** Photopolymerisation of AdECM using Ru/SPS and visible light (400–450 nm).

Towards establishing an adipose tissue-specific biomaterial formulation which mimics the ECM composition of native breast tissue, porcine adipose tissue was decellularised using SDS and isopropanol to remove cellular material and lipids, respectively (Fig. 4a). The resulting AdECM could be photocrosslinked with visible light through incorporation of photoinitiators (0.5 mM Ru and 5 mM SPS) and irradiation at 400–450 nm for 3 min (Fig. 4b), as previously described [53,77]. Tissue-specific ECM from porcine cornea and heart tissue exhibited a reduction in tyrosine groups and an increase in fluorescence after photocrosslinking using this method, highlighting the formation of dityrosine crosslinks within dECM hydrogels [53].

**Fig. 5:**
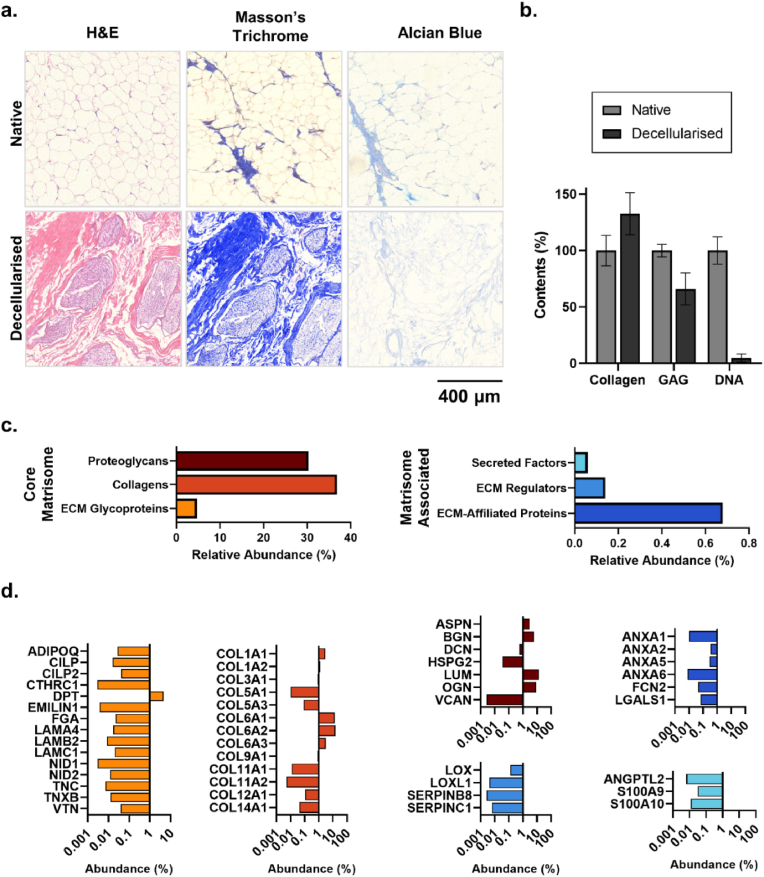
Compositional characterisation of AdECM. **a.** Histological evaluation of tissue structure – H&E, collagens – Masson's Trichrome, and glycosaminoglycans (GAGs) – Alcian Blue compared with native adipose tissue. **b.** Biochemical evaluation of collagen, GAG and DNA contents compared with native adipose tissue. Error bars denote ± standard deviation of the mean. **c.** Relative abundance of core matrisome and matrisome-associated proteins detected in AdECM. **d.** Relative abundance of matrisome proteins, categorised by protein subgroup (colours as per panel c)). Data is presented in log scale, where low and high abundance proteins are presented on the left and right side of the baseline, respectively. (For interpretation of the references to color in this figure legend, the reader is referred to the Web version of this article.)

To verify effective decellularisation of the AdECM, the presence of collagens and glycosaminoglycans (GAGs) was assessed histologically and biochemically, and the ECM composition (i.e. the matrisome) was characterised using liquid chromatography coupled to mass spectrometry (LC-MS). There was a distinct change in tissue structure after decellularisation, with an increased density of ECM and absence of adipocytes, nuclei and cell debris (Fig. 5a). There was a 33% increase in collagen content, while GAGs were reduced by 34% compared to the native tissue (Fig. 5a and b). Importantly, there was 95% reduction in DNA content after decellularisation, validating effective removal of cellular contents (Fig. 5b). The decellularisation process was effective considering that only 26 ± 19.5 ng of DNA remained in the decellularised product, which is below the 50 ng/mg DNA previously defined in decellularisation protocols [86,96]. Furthermore, these data align with previous reports of adipose tissue decellularisation, whereby collagen content increases with removal of cellular components from native tissue, while more-soluble GAGs are reduced due to treatment with surfactants [44,86].

Despite the reduction in GAGs measured, proteomic analyses detected 36 core matrisome proteins and 11 matrisome-associated proteins, with the highest relative abundance attributed to collagens and proteoglycans, at 36.9% and 30.3% respectively (Fig. 5c). The top most abundant proteins (>1% relative abundance) were collagen VI (COL6A1, COL6A2, COL6A3), lumican (LUM), osteoglycin (OGN), biglycan (BGN), dermapontin (DPT), collagen I (COL1A1, COL1A2) and asporin (ASP) (Fig. 5d). The high abundance of collagen VI is characteristic of adipose tissue, playing a key role in anchoring adipocytes to the supportive basement membrane, and demonstrates the specificity of the isolated AdECM [48]. Furthermore, the matrisome proteins identified in the AdECM were similar to those previously detected in decellularised murine mammary glands, however the abundance of the proteins greatly differed, with the AdECM containing a >4-fold greater abundance of proteoglycans and ECM glycoproteins, and 2.5-fold less collagens than the murine dECM [44]. These differences can likely be attributed to a disparity in donor tissue species and the use of a harsher decellularisation protocol which may have diminished more soluble proteoglycans [44]. Nevertheless, these data demonstrate that the isolated AdECM contained a wide range of matrisome proteins from all of the protein subgroups in stoichiometric ratios reflective of native adipose tissue; and therefore, provides appropriate adipose tissue-specific biochemical cues to mimic the breast tumour microenvironment.

### 3.2 Hydrogel characterisation and mechanical profiling

**Fig. 6:**
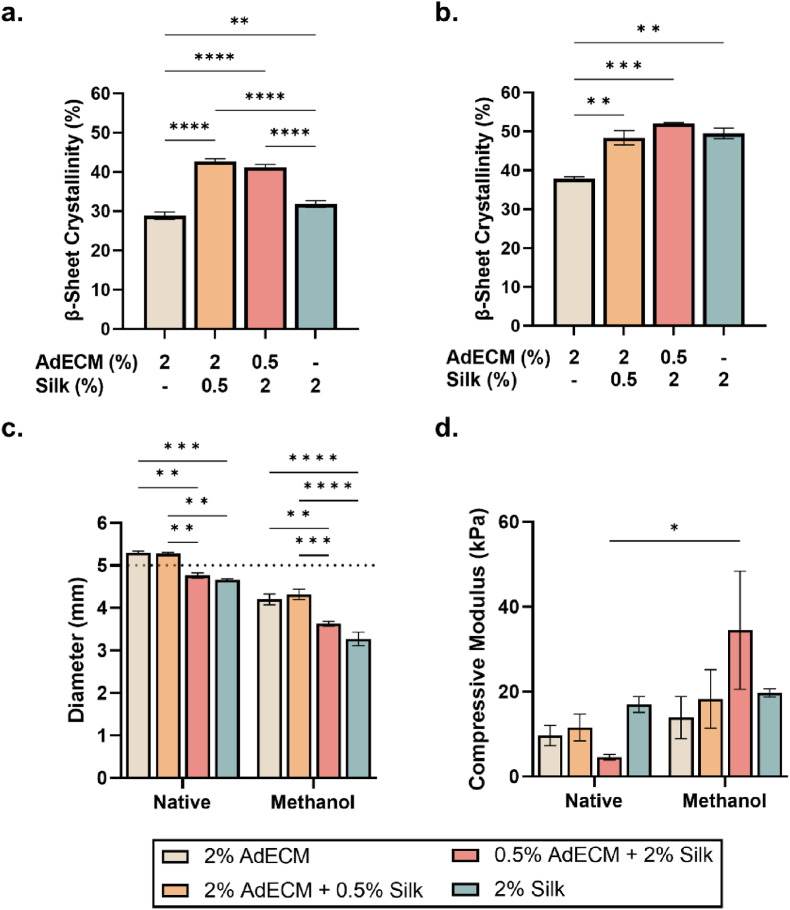
Protein structural conformation and mechanical properties of AdECM/silk hydrogel compositions. β-Sheet crystallinity of **a.** native, and **b.** methanol-treated hydrogels. **c.** Mean diameter and **d.** compressive modulus (kPa) of native and methanol-treated hydrogels. Dotted line shows size of casting mould. Error bars represent ±standard error. Asterisks denote significant differences, *p < 0.05, **p < 0.01, ***p < 0.001, ****p < 0.0001.

To program temporal stiffening into the composite biomaterial, the isolated AdECM was photocrossslinked into hydrogels with silk fibroin in a 4:1 and 1:4 ratio (AdECM:silk fibroin), and the mechanical profile and protein structure characterised (Fig. S1). To understand the potential for β-sheet formation of silk fibroin when blended with the AdECM, hydrogels were methanol treated to weaken protein-protein hydrophobic interactions and stimulate formation of β-sheet secondary structures [78,97]. In native hydrogels (non-methanol treated) there was a significant increase in β-sheet crystallinity (∼10%) in the composite hydrogels compared to AdECM or silk hydrogels alone (P < 0.0001; Fig. 6a; Figs. S2a and c). As expected, silk had a 3% greater β-sheet crystallinity compared with AdECM alone (P < 0.01), and demonstrated similar crystallinity profiles to previously reported silk hydrogels crosslinked both enzymatically and using visible light (Fig. 6a; Figs. S2a and c) [78]. Methanol-treated hydrogels also had ∼10% increase in β-sheet crystallinity compared with native hydrogels highlighting the formation of secondary structures (Fig. 6a and b; Figs. S2b and d) [78,97]. There was a significant increase in β-sheet crystallinity (∼10%) in all hydrogels containing silk, compared to AdECM-only hydrogels (P = 0.001; Fig. 6b; Figs. S2b and d). Interestingly, while the 0.5% AdECM/2% silk hydrogels had the highest β-sheet crystallinity, there were no significant differences compared with 2% AdECM/0.5% silk and 2% silk-only conditions (Fig. 6b). Nevertheless, the presence of β-sheets in AdECM can be explained by the large ECM glycoproteins present within the AdECM (e.g., laminin) which contain β-sheet structures and may have caused further protein-protein interactions and enhanced crystallinity within the composite material blends [98].

The size of the hydrogels in all compositions was not affected by the formation of β-sheets. However, hydrogels containing 2% AdECM had a significantly larger diameter in comparison with compositions containing 2% silk, in both native and methanol-treated samples (P < 0.0001; Fig. 6c). Methanol treatment resulted in reduced diameters in all groups, likely due to the weakening of hydrophobic protein-protein interactions and strengthening of hydrogen bonds during the formation of β-sheet secondary structures [99] (Fig. 6c and d). While there was an increase in compressive modulus in all compositions in response to methanol treatment, a significant increase was only detected in the 0.5% AdECM/2% silk composition (∼30 kPa) (P < 0.05; Fig. 6d). Given that there was no significant difference in β-sheet crystallinity between other silk-containing hydrogels, the mechanical properties observed may be the result of complex interactions or molecular entanglement between the AdECM and silk fibroin which result in alternative structural conformations. Furthermore, the variability in compressive modulus highlights the variation in spontaneous interactions between the AdECM and silk which have not previously been characterised. In support of this, no stiffening occurred in the 2% silk composition (Fig. 6d), suggesting that AdECM may enhance the formation of secondary structures which have been previously shown to be restrained following photocrosslinking [78].

**Fig. 7:**
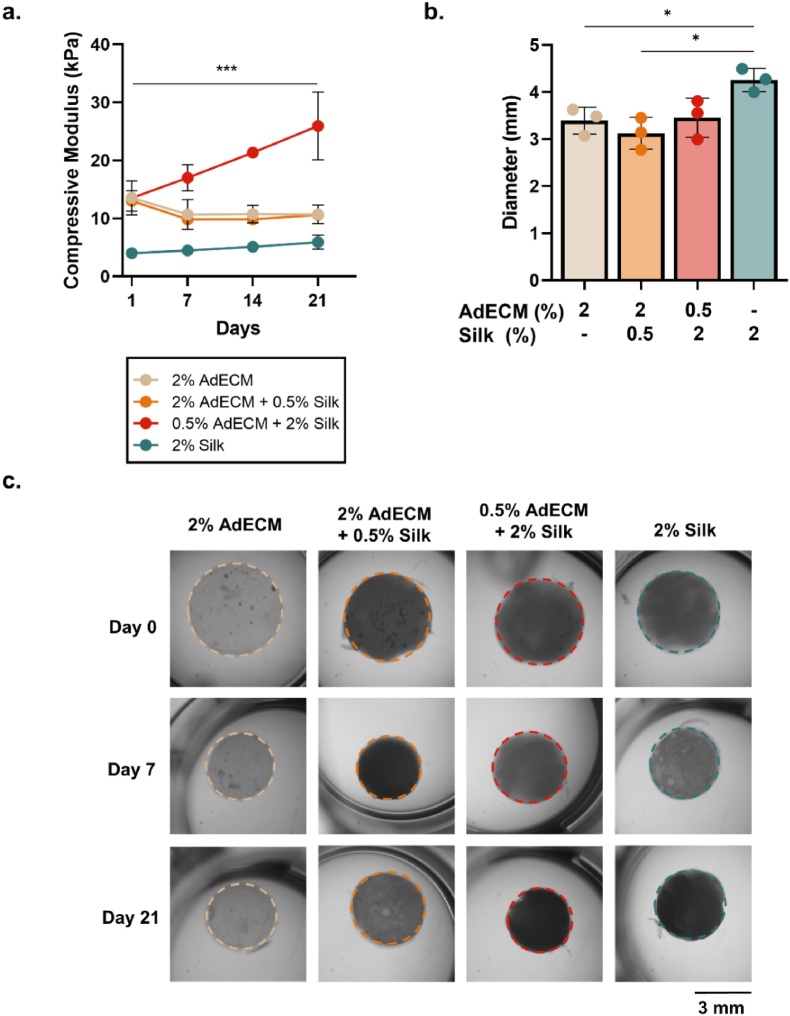
Longitudinal changes in mechanical and structural properties of AdECM/silk hydrogel compositions. **a.** Compressive modulus (kPa) and **b.** mean diameter of hydrogels incubated in DMEM over 21 days. Error bars represent ±standard error. Asterisks denote significant differences at each time point, *p < 0.05, ***p < 0.001. **c.** Macro images of hydrogels at day 0, 7 and 21.

To further understand the temporal scale at which these changes in material stiffness occur, the compressive modulus was assessed longitudinally over 21 days. Only one of the four experimental formulations exhibited temporal stiffening properties. There was a significant increase in the compressive modulus of the 0.5% AdECM/2% silk composition from day 1 to day 21, with a final compressive modulus of 26 kPa (P < 0.0001; Fig. 7a; Fig. S3). However, the final compressive modulus of the samples did not reach the stiffness of methanol-treated samples at 34.5 kPa, suggesting that further material stiffening may occur over longer time periods, or, that there is a disparity between the use of a chemical stimulant or self-arrangement to form protein secondary structures (Fig. 6d; Fig. 7a). The duration of material stiffening was consistent with previous studies comparing enzymatic and visible-light crosslinking, where increases in compressive modulus were evident at 2 weeks, with high variation detected [78]. As in the methanol-treated samples, stiffening did not occur in any other composition; however, unlike the methanol-treated samples, the changes in mechanical properties observed did not result in greater reductions in hydrogel diameter (Fig. 7; Fig. S3; Fig. S4a). After 21 days, the 2% AdECM hydrogels had a significantly smaller diameter than the 2% silk hydrogels, suggesting a degradation or loss of loosely-crosslinked AdECM over time (P = 0.0148; Fig. 7b and c). This highlights the likelihood that 2% silk adds both dynamic stiffening properties to AdECM, and provides increased shape stability to the hydrogel, permitting high-resolution photopatterned structures (Fig. S5). Together, these results indicate that interactions between 0.5% AdECM and 2% silk result in dynamic stiffening within the composite material over three weeks despite photocrosslinking, and ultimately mimics the temporal mechanical properties of breast tumours throughout a small region of the pathological domain [2,78].

### 3.3 Phenotypic screening of encapsulated breast cancer cells

**Fig. 8:**
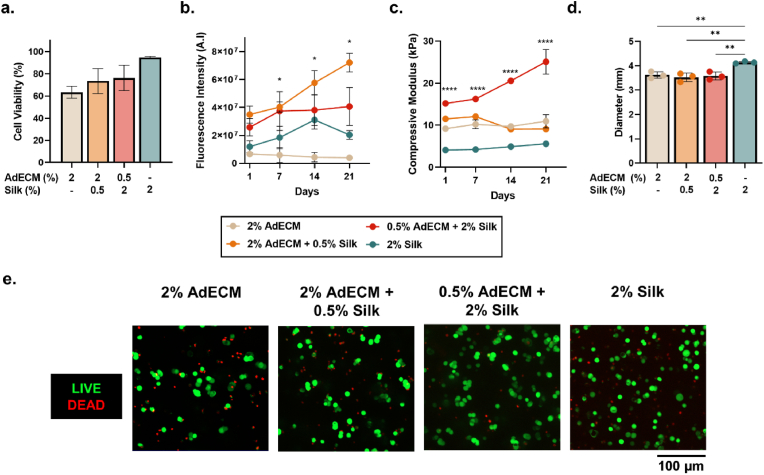
MCF-7 breast cancer cell encapsulation within AdECM/silk hydrogels. **a.** Cell viability 24 h after encapsulation within biomaterials. **b.** Metabolic activity and **c.** compressive modulus over 21 days in culture. **d.** Diameter of cell-laden hydrogels after 21 days. **e.** Representative images of viability staining after 24 h. Calcein-AM (green) and propidium iodide (red) were used to stain live and dead cells respectively. Error bars represent ±standard error. Asterisks denote significant differences at each time point, *p < 0.05, **p < 0.01, ****p < 0.0001. See Table S1and Table S2 for statistical details. (For interpretation of the references to color in this figure legend, the reader is referred to the Web version of this article.)

To assess whether the dynamic temporal cues within the ECM hydrogel formulation induced a malignant phenotype, luminal MCF-7 cells were encapsulated within the AdECM/silk biomaterials and cultured over 21 days. This non-invasive, luminal MCF-7 breast cancer cell line was selected to allow for subtle changes in phenotype to be detected. There were no significant differences in cell viability after encapsulation and photopolymerisation of hydrogels; however, compositions with silk had increased viability by at least 10%, with the 2% silk-only composition demonstrating 95% viability (Fig. 8a–e). These data are consistent with previous studies in AdECM/silk compositions where viability increased with silk addition [57], and suggests that the addition of tyrosine groups available within the material for photocrosslinking (∼5% tyrosine residues in silk fibroin) plays a role in preventing oxidative damage from photopolymerisation [75,100].

The growth patterns of the MCF-7 cells differed between material composition over the three-week study, highlighting the importance of temporal ECM cues in programming tumour cell metabolism. While there was no significant difference in metabolic activity one day after encapsulation, both the AdECM/silk compositions had a significantly higher metabolic activity at day 7, 14 and 21 compared with AdECM-only hydrogels (P = 0.0039; Fig. 8b; Table S1). The metabolic activity of cells within the 2% AdECM/0.5% silk hydrogel increased throughout the study, while the 0.5% AdECM/2% silk hydrogels plateaued from day 7 (Fig. 8b). Similar trends were seen in 2% silk-only hydrogels, while 2% AdECM-only hydrogels maintained a low metabolic activity throughout the whole study (Fig. 8b). These observations suggest that silk increases cellular metabolism within photocrosslinked AdECM, by providing a structural framework and reducing cellular damage, while differences identified between composite materials may be the result of changes in material mechanical properties.

To evaluate this hypothesis and determine whether cell encapsulation affected dynamic stiffening, the compressive modulus of hydrogel compositions containing MCF-7 cells was measured longitudinally across the study. The longitudinal mechanical profile of the cell-encapsulated hydrogels mimicked cell-free conditions, where dynamic stiffening occurred only in the 0.5% AdECM/2% silk composition, reaching a similar stiffness to cell-free hydrogels, of 25 kPa (Fig. 8c). The augmented mechanical properties in the 0.5% AdECM/2% silk hydrogels did not cause a change in diameter compared with other AdECM hydrogels, suggesting that dynamic stiffening occurred without material contraction (Fig. 8d; Fig. S4b). The diameter of the silk-only hydrogels was significantly larger than all other compositions at day 21, reflecting similar trends to cell-free hydrogels (P < 0.0022; Fig. 8d). Interestingly, there was a significant difference in compressive modulus between compositions across the study, with a small reduction in the mechanical properties of 2% AdECM-containing hydrogel compositions (P < 0.0001; Fig. 8c; Table S2). The encapsulated cells may have reduced the amount of crosslinking within the hydrogel, resulting in reduced mechanical properties compared to cell-free hydrogels [100,101]. Nevertheless, these data demonstrate that dynamic stiffening of the composite AdECM/silk formulation was not affected by encapsulation of cells and that the changes in mechanical properties likely altered the cellular metabolic activity [22].

**Fig. 9:**
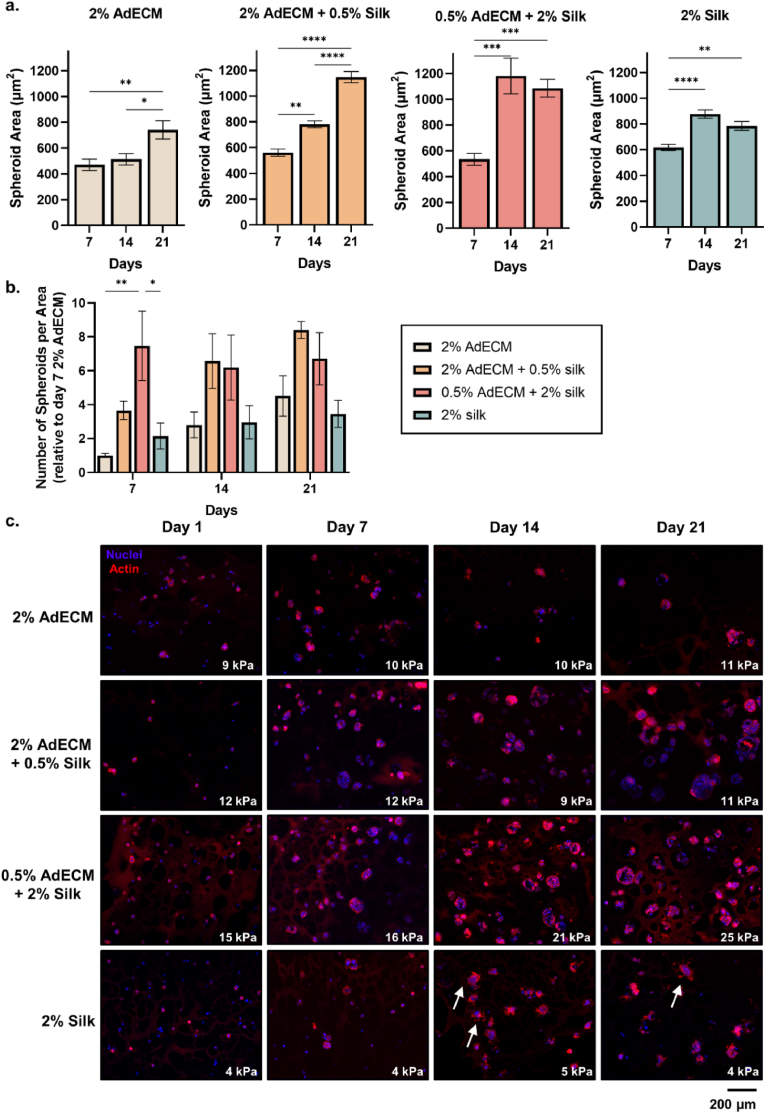
MCF-7 spheroid phenotype progression over 21 days in AdECM/silk hydrogels. **a.** Average spheroid size, **b.** number of spheroids per field-of-view, and **c.** representative immunofluorescence images of actin (red, phalloidin) and nuclei (blue, DAPI). White arrows indicate cell protrusions and the compressive modulus of each hydrogel composition at each time point is noted. Representative images are provided from three replicate biological experiments, each containing three hydrogel technical replicates with triplicate fields-of-view obtained from each. Error bars represent ±standard error. Asterisks denote significant differences, *p < 0.05, **p < 0.01, ***p < 0.001, ****p < 0.0001. (For interpretation of the references to color in this figure legend, the reader is referred to the Web version of this article.)

The viability and growth profiles of the encapsulated MCF-7 spheroids, corresponded to the size, number and morphology of spheroids which grew from day 7 (Fig. 9). AdECM within the composite hydrogels promoted a rounded, spheroid morphology with distinct borders, while spheroids grown in the softer 2% silk-only composition had a less-organised structure, with clear membrane protrusions out from the spheroid, potentially due to the inert material properties which often require biofunctionalisation to improve cellular compatibility and prevent apoptosis (Fig. 9c) [85]. Spheroids grown in 2% AdECM-only grew slowly, with a significant increase in spheroid area detected only at day 21 compared with day 7 and 14 at ∼740 μm^2^ (P = 0.0013; Fig. 9a–c). There was a significant stepwise increase in spheroid area over time for the 2% AdECM/0.5% silk composition, which reached an area of ∼1150 μm^2^ – a 55% increase from 2% AdECM-only composition (P < 0.0001; Fig. 9a–c). This supports the notion that the addition of silk may have provided a structural scaffold for the AdECM and prevented oxidative damage to the cells [75,100].

Comparable trends in spheroid area were detected in the compositions containing 2% silk, where there was a significant increase from day 7–14, however no further increases in spheroid size was seen at day 21 (0.5% AdECM/2 % silk: P < 0.0001; 2% silk: P < 0.0001; Fig. 9a–c). The only dynamic stiffening formulation (0.5% AdECM/2% silk) had the largest spheroid size (1180 μm^2^) by day 14, and contained a significantly larger number of spheroids at day 7 compared with the 2% AdECM- and 2% silk-only compositions; >6-fold and >5-fold difference respectively (P = 0.0003; Fig. 9b). However, while the number of spheroids in the 2% AdECM-only, 2% AdECM/0.5% silk and 2% silk-only compositions grew over the 21 days, by 3.5-fold, 4.8-fold and 1.3-fold respectively, the 0.5% AdECM/2% silk composition retained a similar number of spheroids over time (Fig. 9b). These growth patterns observed only in the dynamic stiffening formulation, along with the metabolic activity data, suggest that the dynamic increase in mechanical properties over culture may have hindered the growth of the MCF-7 spheroids, as seen commonly in normal breast epithelial cells when grown in 3D platforms [11,102]. Previous studies of malignant cells in natural biomaterials have shown an increase in cell proliferation and spheroid growth with increasing mechanical properties, however these studies were restricted by the range of mechanical properties investigated, often reaching only 1–2 kPa [17,62,65]. The 20–25 kPa hydrogels formed in this study likely placed a physical restriction on the outgrowth of cells, as in synthetic PEG hydrogels where spheroid growth increased from 0.2 to 1 kPa but reduced in size in 26 kPa hydrogels [22]. Nevertheless, in the present study, spheroids grown within the 2% silk-only hydrogels (∼5 kPa) were ∼400 μm^2^ smaller in area than the 0.5% AdECM/2% silk hydrogels (∼25 kPa) and morphologically demonstrated invasive cell protrusions from spheroids, supporting previous reports of mechano-regulation driving an invasive and tumourigenic phenotype (Fig. 9b and c) [59,103,104].

### 3.4 Assessment and progression of disease state

**Fig. 10:**
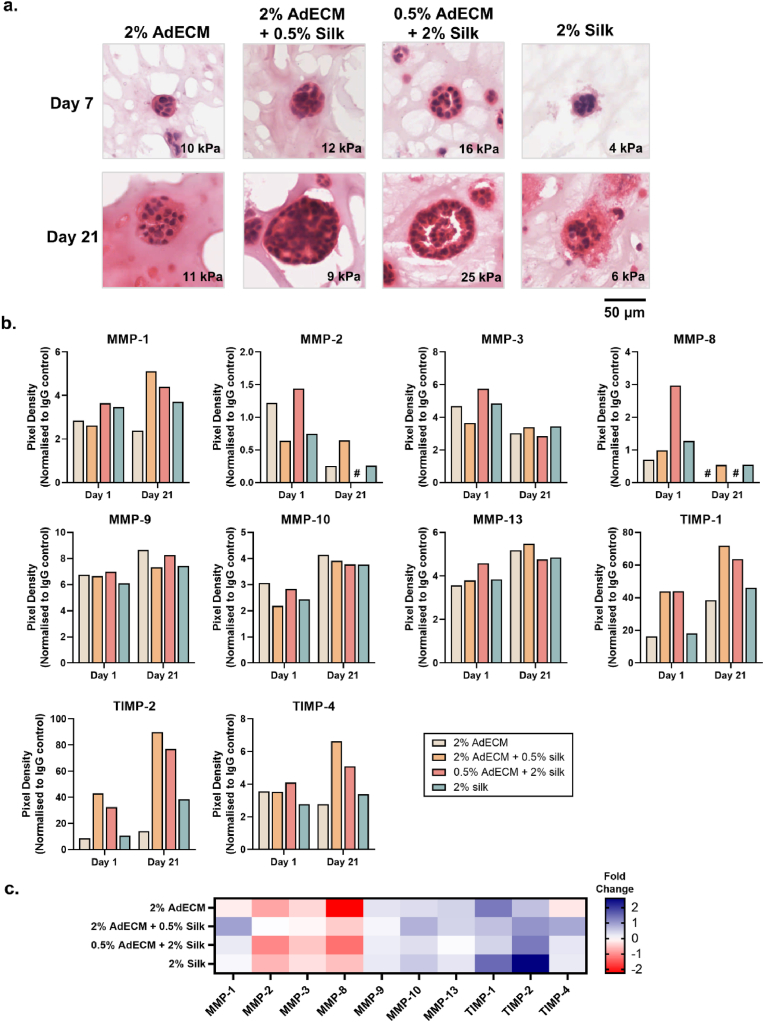
Assessment of MCF-7 spheroid structure and ECM dynamics in AdECM/silk hydrogels. **a.** Representative images at 20 × magnification of haematoxylin and eosin stained sections at day 7 and day 21. The compressive modulus of each hydrogel composition is noted. Representative images are provided from three replicate biological experiments, each containing three hydrogel technical replicates with triplicate fields-of-view obtained from each. **b.** Quantification of matrix metalloproteinase (MMP) and tissue inhibitor metalloproteinase (TIMP) enzymes secreted at day 1 and day 21. **c.** Fold-change in MMP and TIMP expression between day 1 and day 21.

As breast cancer cells are known to exhibit phenotypic plasticity during tumour progression [11,105,106], a comprehensive investigation of secreted matrix remodelling enzymes, cell-surface markers and ECM production was undertaken to evaluate the suitability of the temporal stiffening ECM hydrogel formulation for modelling tumour progression. The histological structure of spheroids was distinct between compositions at both day 7 and day 21 (Fig. 10a). The 2% AdECM-only composition appeared to have a higher density of ECM with a small number of poorly organised nuclei, while the AdECM/silk composites formed high-cell density spheroids as in native tumours (Fig. 10a). In the only temporally-stiffened 0.5% AdECM/2% silk composition, there was a reduction in ECM within the spheroids, with a highly organised cell border encapsulating a developing lumen, while the 2% silk condition had poor cellular organisation and ECM which protruded beyond the spheroid border (Fig. 10a). Clearly, the AdECM provided a necessary matrix for forming large spheroid cultures which was not obtained in silk-only formulations. Furthermore, these data suggest that the temporally-stiffened 0.5% AdECM/2% silk hydrogels supported the morphology of normal breast epithelium from day 7, containing highly-organised cells as seen *in vivo* ^107^*,* and may provide a more differentiated and physiological model of breast cancer [107,108].

Assessment of the abundance of secreted matrix metalloproteinase (MMP) and tissue inhibitor metalloproteinase (TIMP) enzymes provided compounding evidence to support the increased spheroid size and number in the compositions containing both AdECM and silk (Fig. 10b and c; Fig. S6). The most highly abundant enzymes in all compositions at each time point were TIMP-1 and TIMP-2 enzymes, while MMP-2 and MMP-8 were the lowest (Fig. 10b). TIMP-1 expression was highest in the AdECM/silk compositions and has been shown to increase cell proliferation, which may suggest a transition to a more aggressive phenotype as in triple-negative breast cancer [109]. Furthermore, MMP-1 and MMP-8 secretion was increased in the 2% AdECM/0.5% silk composition between day 1 and day 21, and at a lesser extent for the temporally-stiffened 0.5 % AdECM/2% silk composition (Fig. 10b and c). These MMPs are collagenases which assist in remodelling the ECM by cleaving collagen I and collagen III and have been shown to increase cell proliferation and metastasis in breast cancer [110,111]. The reduced expression of MMP-1 and MMP-8 in the temporally-stiffened 0.5 % AdECM/2% silk composition on day 21 was therefore expected, considering that spheroid growth was arrested from day 14, potentially due to enhanced physical restriction imparted from silk-AdECM interactions (Fig. 9b; Fig. 10b and c). TIMP-4 secretion in the AdECM/silk compositions may further correlate with the larger spheroid size observed, considering that TIMP-4 is implicated in malignant progression (Fig. 10b and c) [112]. Furthermore, MMP-2 was downregulated in all groups and further suppressed by increased TIMP-2 secretion, which may also have permitted spheroid growth longitudinally (Fig. 10b and c). Taken together, these data suggest that greater matrix remodelling occurred generally in AdECM/silk composite hydrogels, which may explain the enhanced spheroid growth profiles. This suggests that the addition of AdECM is important to mimic the matrix remodelling profiles of the native tumour microenvironment, allowing for greater tumour growth and ECM remodelling during tumour progression.

**Fig. 11:**
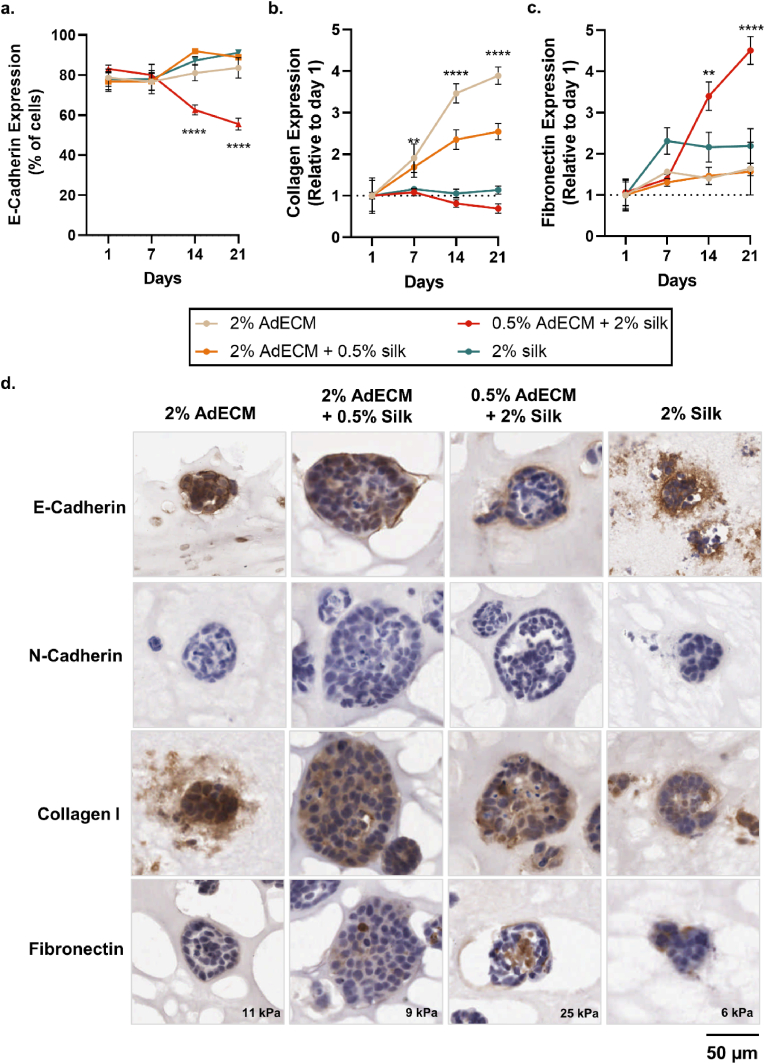
Epithelial-mesenchymal transition and extracellular matrix production of MCF-7 cells in AdECM/silk hydrogels. **a.** E-cadherin, **b.** collagen I and **c.** fibronectin expression over 21 days in culture. Error bars represent ±standard error. Asterisks denote significant differences at each time point, **p < 0.01, ****p < 0.0001. See Table S3, Table S4 and Table S5 for statistical details. **d.** Representative immunohistochemistry images of E-cadherin, N-cadherin, collagen I and fibronectin. The compressive modulus of each hydrogel composition is noted. Representative images are provided from three replicate biological experiments, each containing three hydrogel technical replicates with triplicate fields-of-view obtained from each.

With clear changes in matrix remodelling capacity over longitudinal culture, the expression of phenotypic markers and ECM proteins was investigated. During tumour progression, cells undergo phenotypic plasticity (e.g., epithelial-mesenchymal-transition (EMT)), which involve remodelling of cell-cell adhesion complexes and morphological changes that result in more motile cells which can break through the basement membrane and disseminate away from the original tumour to colonise secondary sites [105,106,[113], [114], [115]]. Cellular EMT is characterised by a loss of cell-cell adhesion, down-regulation of epithelial genes (such as E-cadherin), up-regulation of N-cadherin and vimentin, and increased migratory capacity [113]. There were no differences in E-cadherin expression at day 1 or day 7 across compositions, with a positive cell population of ∼80% (Fig. 11a). In the 2% silk-only composition, E-cadherin exhibited diffuse staining around the spheroid and was not restricted to the spheroid – potentially demonstrating an apoptotic phenotype due to a lack of material functionalisation (Fig. 9c; Fig. 11d). There was a significant reduction in E-cadherin expression in the temporally-stiffened 0.5% AdECM/2% silk composition on day 14 and day 21, with a 35.5% reduction in E-cadherin-positive cells (P < 0.0001; Fig. 11a–d; Table S3). Generally, the E-cadherin-negative cells were present within the core of spheroids, surrounded by an E-cadherin-positive cell layer (Fig. 11d). This distinct organisation of the MCF-7 cancer cells within the stiffening formulation and the absence of migrating cells suggests that temporal matrix stiffening may delay cancer cell intravasation and dissemination to new sites. The reduction in E-cadherin observed is similar to previous studies, including those on seeded AdECM scaffolds, whereby MCF-7 cells expressed similar levels of adhesion molecules to xenografts over Matrigel™ substrates – including increased N-cadherin and vimentin, as well as reduced E-cadherin [43,116]. However, in the present study, despite reductions in E-cadherin, there was no expression of mesenchymal markers, N-cadherin or vimentin (data not shown), highlighting the utility of this model for only early-stage tumour progression (Fig. 11d). This may indicate that other tumour microenvironment cues, such as hypoxia or cell signalling, may be required to induce the complete EMT phenotype within this *in vitro* model. However overall, the adipose tissue-specific hydrogel and accompanying temporal stiffening cues described in this study, generated similar breast cancer phenotypes (increased EMT markers) to other *in vitro* hydrogel models without the increased complexity required for *ex vivo* culture of both adipose tissue and breast cancer cells [33].

The expression of ECM molecules in the MCF-7 spheroids showed striking differences across material compositions, indicating the importance of tissue-specific ECM cues for driving cellular processes. AdECM appeared to stimulate the production of collagen I within and around the spheroids, whereby compositions containing 2% AdECM had significantly greater collagen expression than compositions containing 2% silk at each time point (P < 0.0001; Fig. 11b–d; Table S4). While traditionally stromal cells were thought to be responsible for the majority of collagen deposition, proteomic characterisation of human breast tumour ECM signatures revealed that both tumour and stromal cells contribute differentially to forming ECM throughout tumour progression, and that collagen I can be expressed by invasive breast cancer cells [117]. In support of this, key collagenase enzymes (MMP-1 and MMP-8) were downregulated in 2% AdECM hydrogels across the study suggesting a behavioural shift in these cells (Fig. 10b). As collagen I expression was found surrounding the spheroids in the 2% AdECM-only hydrogel, it seems that the AdECM reprogrammed cell behaviour and promoted an ECM deposition regime in the absence of stromal cells, which was not seen in other material compositions (Fig. 11d). This is a novel aspect of this model, considering that compartmentalised fibroblast and cancer cocultures are normally required to model ECM deposition and remodelling *in vitro* [10,39,[118], [119], [120]]. Nevertheless, while this model demonstrates some ECM remodelling by cancer cells, it is important to note that the stiffening in this model occurs by ECM crosslinking without cancer-associated fibroblasts and therefore in the absence of nascent matrix deposition and contraction which predominantly drive stiffening processes in native breast tumours.

Fibronectin is a known mesenchymal marker which is upregulated in invasive breast cancer cells [121]. While all material compositions contained a population of fibronectin-expressing cells, there was a significant upregulation of fibronectin in the temporally-stiffened 0.5% AdECM/2% silk composition compared with other mechanically-static compositions (P = 0.0059; Fig. 11c and d; Table S5). The positive cells were located in the spheroid core, coinciding with the E-cadherin-negative cells (Fig. 11d). It has been shown that mechanical compression can result in an increase in fibronectin expression and the development of an invasive phenotype [59,122], which may explain the upregulation seen in these dynamic stiffening hydrogels. Furthermore, the upregulation of fibronectin within the core of the spheroids demonstrates the potential formation of a cancer stem cell-like population that show attributes of EMT (i.e., loss of E-cadherin, cellular organisation, lumen formation) which could increase their invasive potential [115,116,123,124]. Considering the luminal, acinar structure of these spheroids, this programmable AdECM/silk fibroin hydrogel may aid in the development of differentiated and heterogeneous breast cancer spheroids which mimic the phenotypic transition from an *in situ* carcinoma to an invasive carcinoma phenotype through the upregulation of fibronectin and subsequent promotion of EMT programs (Fig. 1; Fig. 11a).

Taken together this model provides a number of novel aspects to preclinical breast cancer models, without the requirement of complex cocultures. The temporal dynamic stiffening achieved in one of the formulations resulted in the growth arrest of spheroids and a maintenance of metabolic activity after 14 days, which contradicts many studies demonstrating greater cell proliferation in stiffer hydrogels [17,62,65]. However, unlike previous static mechanical models [58,64,68,69], the gradual material stiffening achieved in the 0.5% AdECM/2% silk composition resulted in distinct phenotypic changes which may be more reflective of the heterogeneity of physiological tumours. The breast cancer spheroids self-organised into organised tumouroids with acinar-like structures as seen in breast epithelium *in vivo* [11,125]. Interestingly, these breast tumouroids contained a central aggregate of cells which expressed a more mesenchymal and aggressive phenotype, highlighting the local heterogeneity present within the spheroids. This phenotypic heterogeneity is important to incorporate within preclinical models considering that intra-tumoural heterogeneity is a key clinical challenge identified for effective treatment of patients [126]. This has important implications for future 3D *in vitro* drug discovery studies, given that this model can be scaled into a high-throughput format and generates heterogenous, stimuli-responsive spheroids which mimic the complexity of mature breast tissue. Finally, this material system and model have increasing relevance moving forward, as while porcine adipose tissue was used here for large-scale and reliable harvesting, there is now a clear opportunity to create distinct human-based models by harvesting adipose tissue and breast cancer cells from matched patients.

## 4 Conclusion

Replicating the dynamic plasticity of disease microenvironments is a distinct challenge when developing accurate preclinical models, due to intricate cell interactions and enduring changes to the interstitial ECM. In this study, a composite biomaterial formulation was developed which uncouples tissue-specific ECM changes from the complex cellular signalling that occurs in coculture models, using breast cancer as an example. AdECM and silk fibroin were photocrosslinked using visible light to generate a biomaterial which replicates the adipose-rich ECM composition of the breast and importantly, in a specific formulation, mimics the progressive increase in mechanical properties during tumour progression over three weeks. The compressive modulus of the resulting cell-encapsulated hydrogel formulation varied ∼10–15 kPa over the study with the spontaneous formation of β-sheet secondary structures. Encapsulated luminal MCF-7 breast cancer cells grew as larger spheroids, and in greater numbers than single or mechanically-static material compositions. Spheroids within the dynamic stiffening matrix, replicated the structure of normal breast epithelium, containing a lumen which housed a population of cancer cells displaying a more advanced phenotype. Upon reaching ∼1000 μm^2^ in area, the spheroids underwent growth arrest, potentially due to mechanical compression, which led to a loss of cell-cell adhesion markers and increased expression of fibronectin. This model establishes intra-tumoural heterogeneity and the instatement of EMT programs within luminal MCF-7 breast cancer cells in response to temporal ECM changes. While stimuli-responsive biomaterials have been developed for tumour modelling which undergo matrix softening and stiffening, those models are static, do not represent the scale of ECM stiffening and require addition of external stimuli to incite the mechanical transition [58,64,68,69]. This new composite biomaterial formulation does not require external stimuli to undergo progressive stiffening and represents a greater range of compressive moduli than previous studies. With the development of visible light crosslinking of unmodified dECM hydrogel materials, this model system is widely tailorable for a range of diseased states (e.g., pancreatic ductal adenocarcinoma, pulmonary fibrosis and neurodegenerative conditions) through combining tissue-specific materials (e.g., decellularised pancreas, lung or brain) with silk fibroin in compositions which reflect native tissue mechanics.

## CRediT authorship contribution statement

**Gretel Major:** Writing – review & editing, Writing – original draft, Investigation, Formal analysis, Conceptualization. **Minjun Ahn:** Writing – review & editing, Methodology, Conceptualization. **Won-Woo Cho:** Writing – review & editing, Methodology, Conceptualization. **Miguel Santos:** Writing – review & editing, Methodology, Investigation. **Jessika Wise:** Writing – review & editing, Validation, Methodology, Data curation. **Elisabeth Phillips:** Writing – review & editing, Validation, Formal analysis, Conceptualization. **Steven G. Wise:** Writing – review & editing, Methodology, Data curation. **Jinah Jang:** Writing – review & editing, Writing – original draft, Resources, Methodology, Investigation, Conceptualization. **Jelena Rnjak-Kovacina:** Writing – review & editing, Writing – original draft, Resources, Methodology, Investigation, Conceptualization. **Tim Woodfield:** Writing – review & editing, Supervision, Methodology, Investigation. **Khoon S. Lim:** Writing – review & editing, Writing – original draft, Supervision, Resources, Investigation, Funding acquisition, Formal analysis, Conceptualization.

## Declaration of competing interest

The authors declare that they have no known competing financial interests or personal relationships that could have appeared to influence the work reported in this paper.

## Data Availability

Data will be made available on request.
