# Supplementary material for: Programming temporal stiffness cues within extracellular matrix hydrogels for modelling cancer niches
Source: Mater Today Bio. 2024 Feb 16;25:101004. doi: 10.1016/j.mtbio.2024.101004 (PMC10900776; doi:10.1016/j.mtbio.2024.101004)
Supplement: Multimedia component 1 [file mmc1.pdf]

Supplementary Data

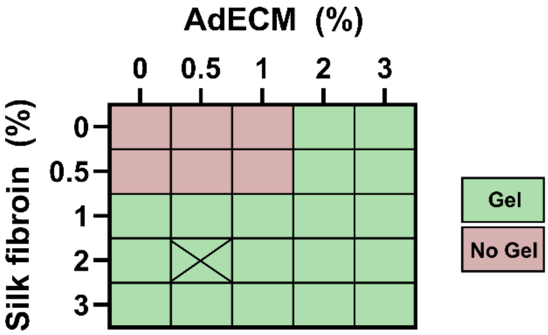

Fig. S1. Overview of AdECM and silk fibroin composite formulations which can be formed into stable hydrogels using a visible light-mediated system. The formulation which demonstrated dynamic stiffening over culture in these experiments is marked with an X.

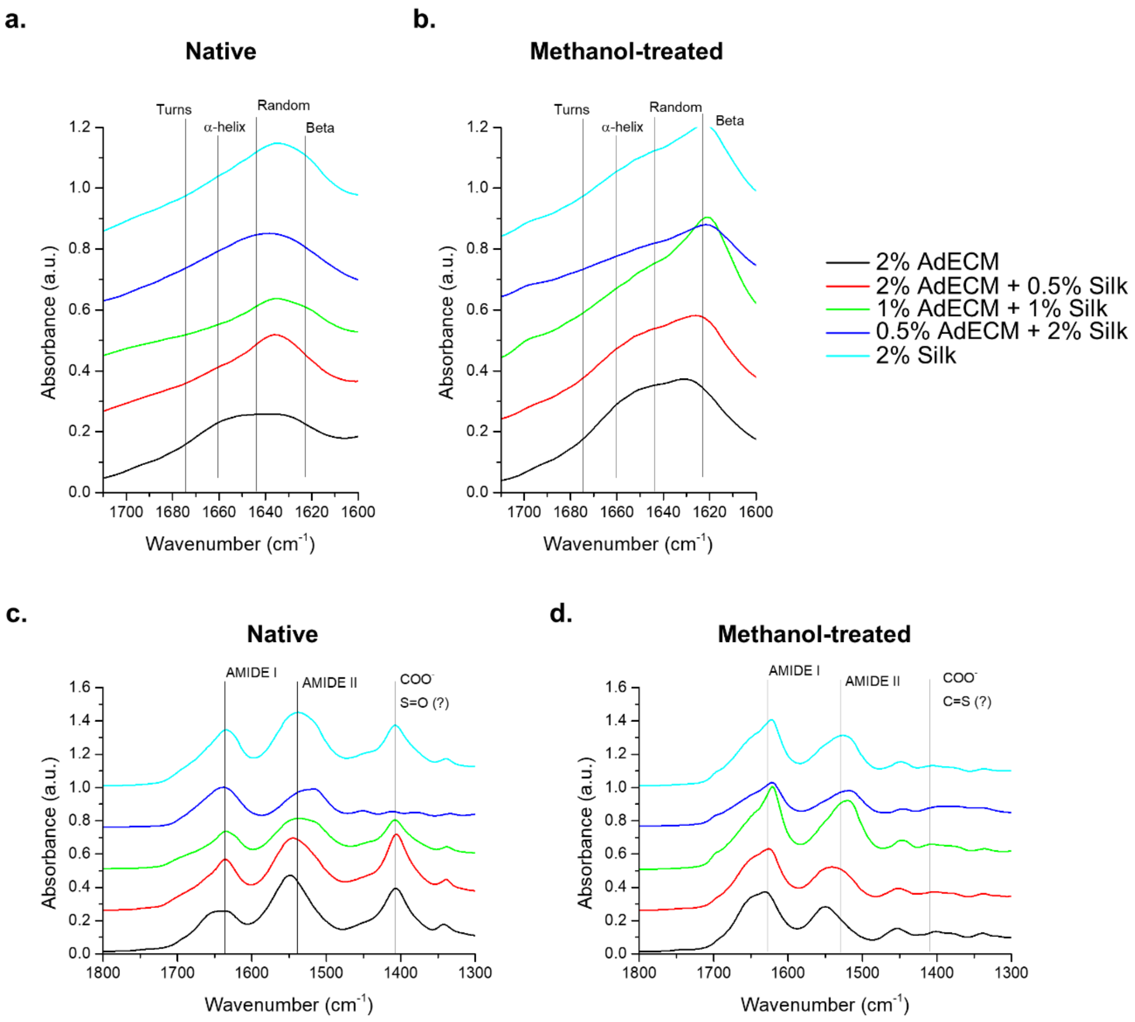

Fig. S2. FTIR scans of AdECM/silk fibroin composite hydrogels. Amide I scan of a. native, and b. methanol-treated samples. Survey scan of c. native, and d. methanol-treated samples

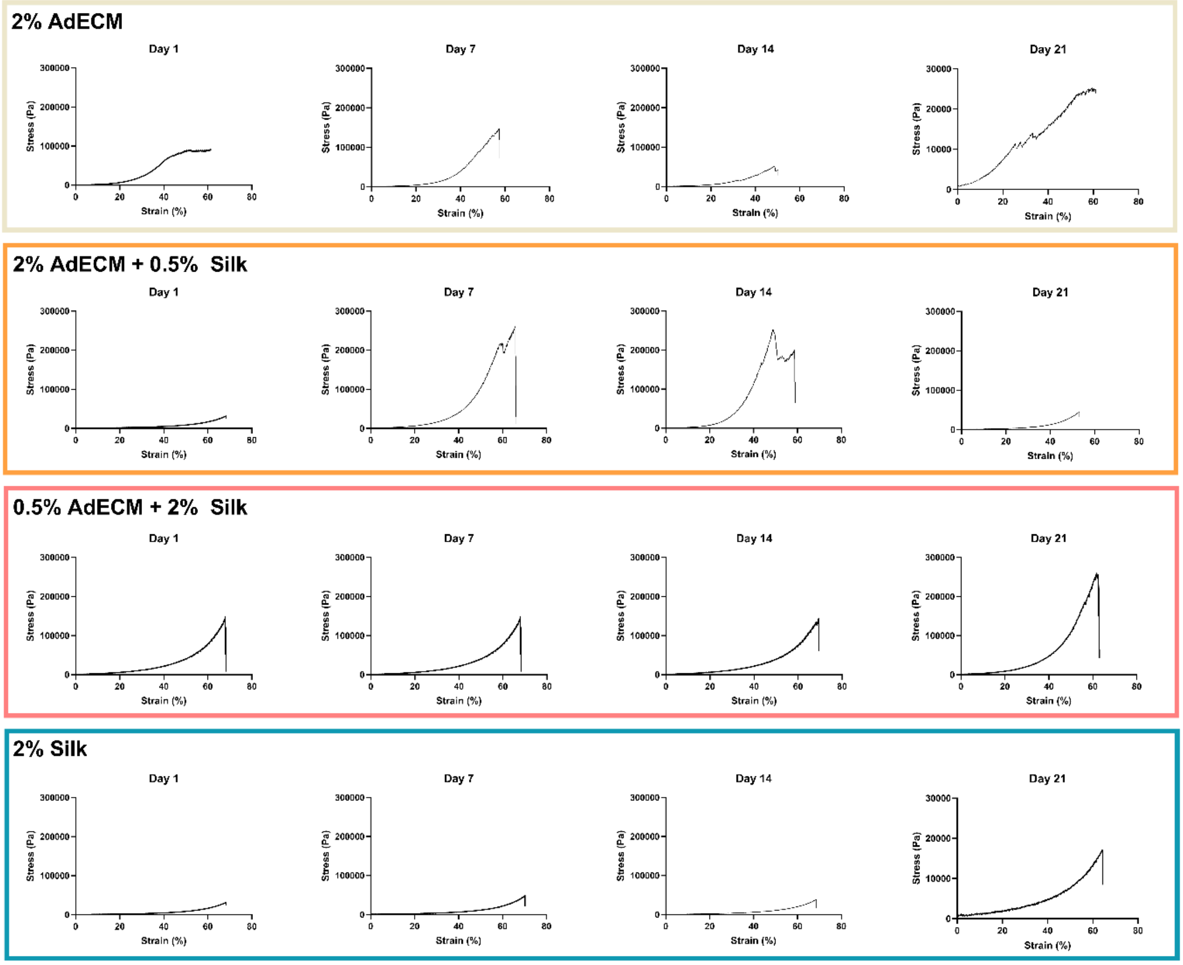

Fig. S3.Stress-strain profiles of AdECM/silk fibroin hydrogel compositions over 21 days.

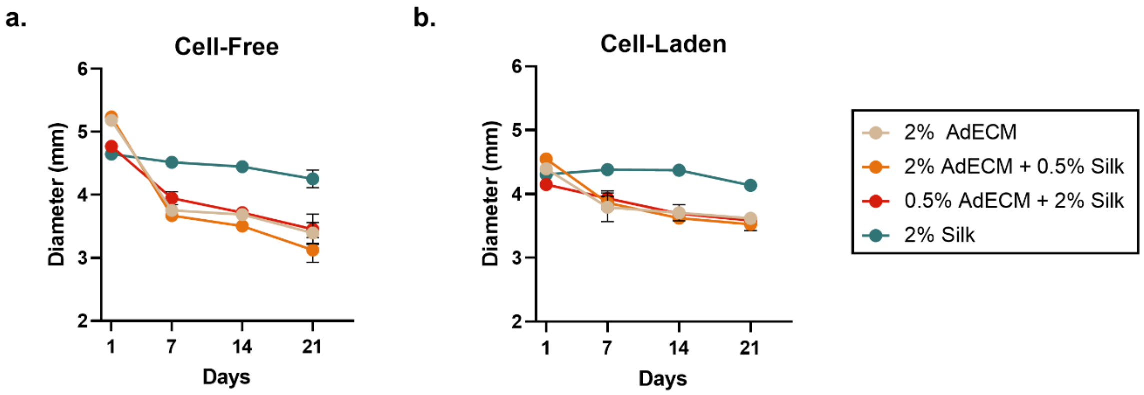

Fig. S4. Mean diameter of hydrogels formed using AdECM/silk fibroin hydrogel compositions over 21 days. **a.** Cell-free hydrogels, and **b.** Hydrogels containing MCF-7 cells. Error bars represent ± standard error.

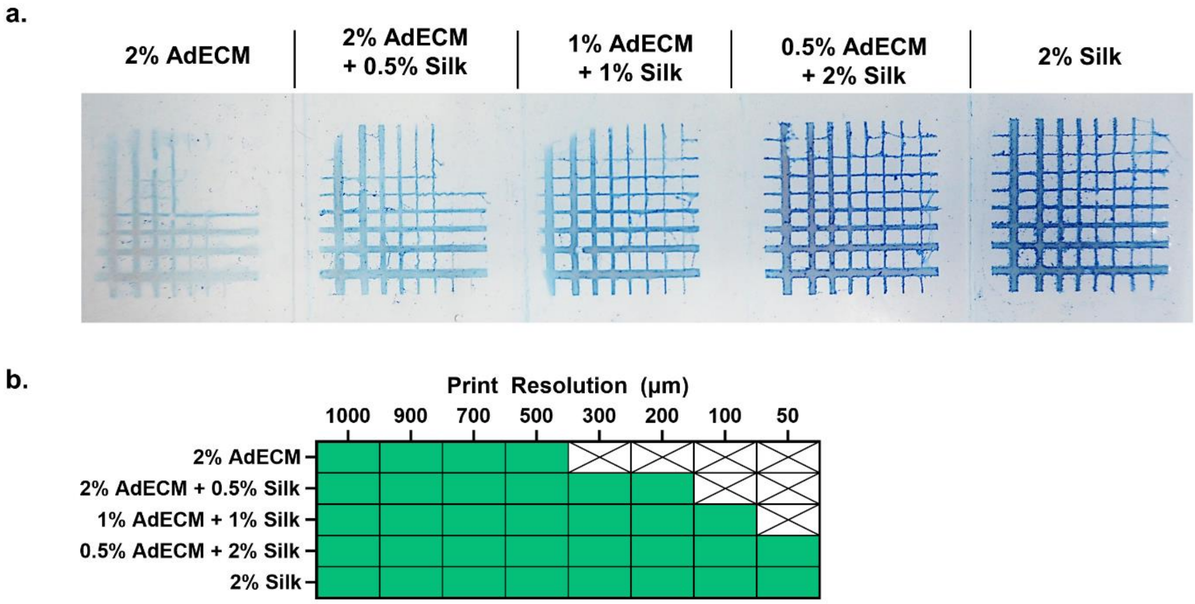

Fig. S5. Photopatterning of AdECM/silk hydrogels using Digital Light Processing. a. Macro images of printed structures stained with Instant*Blue*. **b.** Print resolution evaluation. To assess the print resolution capacity of AdECM/silk fibroin composite materials, materials were photopatterned using a Lumen X printer (CELLINK, USA). The print parameters were as follows, light intensity – 52% (30 mW/cm^2), exposure time – 30 sec/layer, number of layers – 1 and penetration depth – 50 µm. Photopatterned structures were washed in PBS to remove uncrosslinked material and then stained with Instant*Blue* to increase the contrast for imaging.

a.

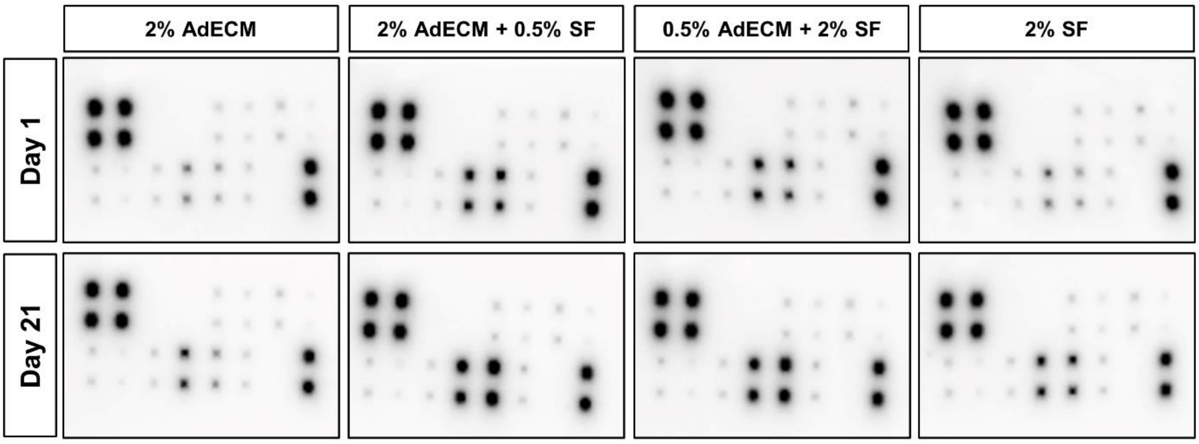

b.

	A	B	C	D	E	F	G	H
**1**	Pos Ctrl	Pos Ctrl	Neg Ctrl	Neg Ctrl	MMP-1	MMP-2	MMP-3	MMP-8
**2**	Pos Ctrl	Pos Ctrl	Neg Ctrl	Neg Ctrl	MMP-1	MMP-2	MMP-3	MMP-8
**3**	MMP-9	MMP-10	MMP-13	TIMP-1	TIMP-2	TIMP-4	Neg Ctrl	Pos Ctrl
**4**	MMP-9	MMP-10	MMP-13	TIMP-1	TIMP-2	TIMP-4	Neg Ctrl	Pos Ctrl

Fig. S6. Human MMP array of media samples collected on day 1 and day 21 from AdECM/silk material compositions. a. Imaged membrane arrays. **b.** MMP membrane key.

Table S1. Significance levels of two-way multiple comparisons following ANOVA, comparing MCF-7 metabolic activity when grown in various AdECM/silk hydrogel compositions. Only significant results are shown.

Significant Comparison	P value
Day 7	
2% AdECM vs. 2% AdECM +0.5% silk	0.0270
Day 14	
2% AdECM vs. 2% AdECM +0.5% silk	0.0445
Day 21	
2% AdECM vs. 2% AdECM +0.5% silk	0.0157
2% AdECM vs. 2% silk	0.0485
2% AdECM + 0.5% silk vs 2% silk	0.0212

Table S2. Significance levels of two-way multiple comparisons following ANOVA, comparing MCF-7 compressive modulus when grown in various AdECM/silk hydrogel compositions. Only significant results are shown.

Significant Comparison	P value
Day 1	
2% AdECM vs. 0.5% AdECM + 2% silk	0.0004
2% AdECM vs. 2% silk	0.0027
2% AdECM + 0.5% silk vs 0.5% AdECM + 2% silk	0.0416
2% AdECM + 0.5% silk vs 2% silk	<0.0001
2% silk vs 0.5% AdECM + 2% silk	<0.0001
Day 7	
2% AdECM vs. 0.5% AdECM + 2% silk	0.0004
2% AdECM vs. 2% silk	0.0004
2% AdECM + 0.5% silk vs 0.5% AdECM + 2% silk	0.0165
2% AdECM + 0.5% silk vs 2% silk	<0.0001
2% silk vs 0.5% AdECM + 2% silk	<0.0001
Day 14	
2% AdECM vs. 0.5% AdECM + 2% silk	<0.0001
2% AdECM vs. 2% silk	0.0048
2% AdECM + 0.5% silk vs 0.5% AdECM + 2% silk	<0.0001
2% AdECM + 0.5% silk vs 2% silk	0.0173
2% silk vs 0.5% AdECM + 2% silk	<0.0001
Day 21	
2% AdECM vs. 0.5% AdECM + 2% silk	<0.0001
2% AdECM vs. 2% silk	0.0016
2% AdECM + 0.5% silk vs 0.5% AdECM + 2% silk	<0.0001
2% silk vs 0.5% AdECM + 2% silk	<0.0001

Table S3. Significance levels of two-way multiple comparisons following ANOVA, comparing MCF-7 E-cadherin expression when grown in various AdECM/silk hydrogel compositions. Only significant results are shown.

**Significant Comparison**	**P value**
Day 14	
2% AdECM vs. 0.5% AdECM + 2% silk	<0.0001
2% AdECM + 0.5% silk vs. 0.5% AdECM + 2% silk	<0.0001
0.5% AdECM + 2% silk vs. 2%	<0.0001
Day 21	
2% AdECM vs. 0.5% AdECM + 2% silk	0.0157
2% AdECM + 0.5% silk vs. 0.5% AdECM + 2% silk	0.0485
0.5% AdECM + 2% silk vs. 2%	0.0212

Table S4. Significance levels of two-way multiple comparisons following ANOVA, comparing MCF-7 collagen I expression when grown in various AdECM/silk hydrogel compositions. Only significant results are shown.

**Significant Comparison**	**P value**
Day 7	
2% AdECM vs. 0.5% AdECM + 2% silk	0.0096
2% AdECM + 0.5% silk vs 0.5% AdECM + 2% silk	0.0463
Day 14	
2% AdECM vs. 0.5% AdECM + 2% silk	<0.0001
2% AdECM vs 2% AdECM + 2% silk	<0.0001
2% AdECM vs. 2% silk	<0.0001
2% AdECM + 0.5% silk vs 0.5% AdECM + 2% silk	<0.0001
2% AdECM + 0.5% silk vs 2% silk	<0.0001
Day 21	
2% AdECM vs. 0.5% AdECM + 2% silk	<0.0001
2% AdECM vs 2% AdECM + 2% silk	<0.0001
2% AdECM vs. 2% silk	<0.0001
2% AdECM + 0.5% silk vs 0.5% AdECM + 2% silk	<0.0001
2% AdECM + 0.5% silk vs 2% silk	<0.0001

Table S5. Significance levels of two-way multiple comparisons following ANOVA, comparing MCF-7 fibronectin expression when grown in various AdECM/silk hydrogel compositions. Only significant results are shown.

Significant Comparison	P value
Day 14	
2% AdECM vs. 0.5% AdECM + 2% silk	0.0048
2% AdECM + 0.5% silk vs 0.5% AdECM + 2% silk	0.0024
0.5% AdECM + 2% silk vs 2% silk	0.0402
Day 21	
2% AdECM vs. 0.5% AdECM + 2% silk	<0.0001
2% AdECM + 0.5% silk vs 0.5% AdECM + 2% silk	<0.0001
0.5% AdECM + 2% silk vs 2% silk	<0.0001
